# *CYP2D6* gene resequencing in the Malagasy, a population at the crossroads between Asia and Africa: a pilot study

**DOI:** 10.2217/pgs-2021-0146

**Published:** 2022-03-01

**Authors:** E Ricky Chan, Rajeev K Mehlotra, Karim A Pirani, Arsene C Ratsimbasoa, Scott M Williams, Andrea Gaedigk, Peter A Zimmerman

**Affiliations:** 1 Cleveland Institute for Computational Biology, Case Western Reserve University, Cleveland, OH 44106, USA; 2 Center for Global Health & Diseases, Case Western Reserve University School of Medicine, Cleveland, OH 44106, USA; 3 Division of Clinical Pharmacology, Toxicology & Therapeutic Innovation, Children’s Mercy Kansas City, Kansas City, MO 64108, USA; 4 University of Fianarantsoa, Fianarantsoa, Madagascar; 5 CNARP (Centre National d’Application de Recherche Pharmaceutique), Antananarivo, Madagascar; 6 Population & Quantitative Health Sciences, Case Western Reserve University School of Medicine, Cleveland, OH 44106, USA

**Keywords:** *CYP2D6*, Madagascar, malaria treatment, next-generation sequencing, *Plasmodium vivax*, primaquine

## Abstract

**Background:**

*Plasmodium vivax* malaria is endemic in Madagascar, where populations have genetic inheritance from Southeast Asia and East Africa. Primaquine, a drug of choice for vivax malaria, is metabolized principally via CYP2D6. *CYP2D6* variation was characterized by locus-specific gene sequencing and was compared with TaqMan™ genotype data.

**Materials & methods:**

Long-range PCR amplicons were generated from 96 Malagasy samples and subjected to next-generation sequencing.

**Results:**

The authors observed high concordance between TaqMan™-based *CYP2D6* genotype calls and the base calls from sequencing. In addition, there are new variants and haplotypes present in the Malagasy.

**Conclusion:**

Sequencing unique admixed populations provides more detailed and accurate insights regarding *CYP2D6* variability, which may help optimize primaquine treatment across human genetic diversity.

Hepatic cytochrome P450 2D6 (CYP2D6) is involved in the metabolism of up to 21% of drugs, many of which are commonly prescribed [1]. It is among the most extensively studied and characterized polymorphic drug-metabolizing enzymes [2], with variation including single nucleotide variants, short insertions and deletions, as well as gene copy number variations (CNVs). The latter encompass deletions of the entire gene, gene duplications and multiplications, and structural rearrangements with the highly similar *CYP2D7* pseudogene [3]. The Pharmacogene Variation Consortium (PharmVar) currently lists more than 140 defined *CYP2D6* haplotypes or ‘star’ (*) alleles [4,5], not counting suballeles or alleles with CNVs. Many of the listed alleles contribute, in part or entirely, to altered rates of CYP2D6-mediated drug metabolism due to increased function, decreased function or the absence of any catalytic activity [2,3].

A method for translating *CYP2D6* genotype to phenotype including the assignment of individuals into four phenotype groups (ultrarapid metabolizers [UM], normal metabolizers [NM], intermediate metabolizers [IM] and poor metabolizers [PM]) has been developed by the Clinical Pharmacogenetic Implementation Consortium [6]. Briefly, a value of 0, 0.25, 0.5 or 1 is assigned to each star allele and the sum of both values determines the activity score (AS) of a genotype/diplotype. AS groups are subsequently translated into phenotype as follows: UM (AS > 2.25), NM (AS = 1.25, 1.5, 2.0 and 2.25), IM (AS = 0.25, 0.5, 0.75 and 1.0) and PM (AS = 0).

Among the large number of drugs that undergo CYP2D6-mediated metabolism are 8-aminoquinoline antimalarials primaquine (PQ) [7–16] and tafenoquine [17,18]. These two drugs are the only known therapies effective against latent malaria caused by *Plasmodium vivax* and *Plasmodium ovale* [19–22]. These malaria parasites have the ability to form dormant liver stages called 'hypnozoites' [23–27] that persist in the liver from weeks to months and cause relapses after clearance of the acute blood-stage infection [23,25,27]. Clinical and laboratory evidence suggest that the efficacy of PQ may depend on genetic variation in *CYP2D6*; vivax malaria relapses following PQ treatment have predominantly been observed in patients carrying *CYP2D6* decreased function alleles and nonfunctional alleles in various combinations giving rise to IM and PM phenotypes [20,28–31].

Recently, for the first time, the authors investigated *CYP2D6* genetic variation in Madagascar [29], an island nation in the Indian Ocean, approximately 400 km (250 miles) off the East African coast. There, vivax malaria is endemic [32], and PQ could be deployed to support efforts toward national malaria elimination [33]. In addition, the Malagasy populations offer a unique, rich admixture history between Southeast Asian and East African ancestral populations and the potential of harboring novel variants and/or combinations of known variants [34–38]. In a recent study by the authors [29], using commercially available TaqMan™ genotyping assays on a custom-designed OpenArray (Thermo Fisher Scientific, MA, USA), 211 samples were genotyped for 29 single-nucleotide polymorphisms (SNPs) and interrogated for the presence of CNVs including the CYP2D6*5 gene deletion, duplications, hybrid genes and tandem arrangements [39–42]. Nine star alleles were detected (CYP2D6*2, *4, *10, *17, *29, *40, *41, *45 and *100). In addition, the CYP2D6*5 gene deletion, gene duplications (CYP2D6*1x2, *2x2, *4x2 and *35x2) and alleles with tandem arrangements (CYP2D6*36 + *10 and *36x2 + *10) were found in the studied Malagasy [29].

In the present study, the authors applied a locus-specific gene sequencing approach to further characterize the *CYP2D6* variation in Madagascar and compare it with the OpenArray genotype data. In the authors’ initial study [29], a discrete set of *CYP2D6* alleles was tested, covering many commonly observed variants across populations; the allele selection was, however, limited, as there are no predesigned TaqMan genotyping assays for numerous variants of potential interest. Being a unique population, it is likely that some of the individuals may have undetected rare or novel alleles, which may or may not be functionally relevant. This limitation may influence the observed frequency of *CYP2D6* alleles, especially the normal function CYP2D6*1 allele, which is assigned if no other SNPs are identified [3]. Other alleles may default to CYP2D6*2 or others, depending on whether they are on a CYP2D6*1-like or *2-like backbone (for further information on ‘default’ assignments of alleles, please see the work of Nofziger *et al.* [3]). Therefore, to validate the *CYP2D6* genotype results [29] and to facilitate the characterization of rare or novel allelic variants, 96 randomly selected Malagasy samples from the initial study were subjected to targeted next-generation sequencing (NGS).

## Materials & methods

### Study protocols, subjects, sample collection & processing

This investigation is a part of the ongoing malaria epidemiological studies being conducted in the western highlands fringe region of Madagascar [32,43–45]. Detailed information about the study sites, subjects, blood sample collection and DNA extraction was provided previously [29,43].

### *CYP2D6* genotyping procedure

In an initial study by the authors [29], 211 samples were genotyped for a total of 29 SNPs, using commercially available TaqMan genotyping assays on a custom-designed OpenArray (Thermo Fisher Scientific). This SNP panel allowed for the identification of 27 star alleles catalogued by PharmVar. In addition, all samples were interrogated for the presence of CNVs including the CYP2D6*5 gene deletion, duplications, hybrid genes and tandem arrangements using qualitative long-range PCR (XL-PCR) and quantitative copy number assays. TaqMan and CNV results informed a sample’s genotype, which from here on is referred to as TaqMan-based genotype calls.

### *CYP2D6* sequencing procedures

#### Next-generation sequencing

From this sample set (n = 211), 96 samples were randomly selected and subjected to Illumina high-throughput sequencing. First, XL-PCR amplicons (6.7 kb) were generated with a *CYP2D6*-specific forward (5′-TCACCCCCAGCGGACTTATCAACC-3′) and reverse (5′-CGACTGAGCCCTGGGAGGTAGGTAG-3′) primer set [46,47]. This XL-PCR amplicon is referred to here, and in previous publications, as Fragment A. XL-PCR products were sheared on a Covaris^®^ instrument to an estimated size of 300 bp. Next, all samples were prepared for sequencing according to the Nextera XT DNA Library Preparation Kit (Illumina Inc., CA, USA). Samples were then sequenced on an Illumina MiSeq multiplex run for 2 × 300 bp paired-end reads.

Illumina MiSeq-generated sequences were first evaluated through FastQC for initial quality control. Sequence reads were trimmed for quality (Q > 20) and adapter sequences using the wrapper script Trim Galore! (www.bioinformatics.babraham.ac.uk/projects/trim_galore/). Reads that passed the quality filter were aligned to the *CYP2D6* reference sequence (RefSeq NG_008376.4) using the Bowtie 2 alignment algorithm [48]. Genotype calls at each base position were determined using SAMtools mpileup [49,50]. Only positions with a coverage of more than 10 reads were considered. SNPs were called a 'homozygous reference' when greater than 70% of reads were ‘reference’ and a 'homozygous variant' when greater than 30% of reads were ‘reference’; SNPs with read frequencies between these two cutoffs were considered ‘heterozygous’ for that position. Nucleotide insertions and deletions (indels) were called by manual visualization of the alignments using the Integrative Genomics Viewer (IGV, https://software.broadinstitute.org/software/igv/).

#### Sanger sequencing

Allele-specific long-range PCR (ASXL-PCR) and Sanger sequencing was utilized to confirm NGS findings and characterize novel haplotypes for one sample. Since no genomic DNA was available for sample 1040105 for further characterization, ASXL-PCR amplicons were generated using the remaining fragment A from the initial study [29]. Primers and PCR reactions were adapted from those published previously [46,51]. Briefly, one allele was amplified with a reverse primer that specifically amplified the allele with a G at position 4482 (rs1135840). For the second allele, forward and reverse primers amplifying the allele with a G at position 842 (rs28371702) were utilized to generate two sets of amplicons, one covering the 5′ and the other the 3′part of the gene. The resulting allele-specific amplicons covered the entire gene including upstream and downstream regions as required by PharmVar for allele designation. Amplicons were subjected to Sanger sequencing using BigDye Terminator chemistry.

#### Sequence annotations

All nucleotide positions throughout this report are based on the current *CYP2D6* RefSeq NG_008376.4 with the ATG start codon being +1, as this is the most commonly utilized coordinate for this gene. Per PharmVar, *CYP2D6* allele definitions contain variants positioned between -1601 and 4482. Although NGS provided data across this region, the final variant calls were limited to the positions between -1100 and 4482 (see the following).

## Results

### *CYP2D6* genotyping

This sample set (n = 96) represents a subset of samples that were previously characterized by TaqMan genotyping and CNV analysis [29]. Among those 96 samples, a total of 34 genotypes were identified, with frequencies ranging from 1.04% (1/96) to 11.46% (11/96). Seven genotypes (CYP2D6*1/*1, *1/*2, *1/*10, *1/*17, *1/*29, *1/*36 + *10 and *36 + *10/*41) were present at frequencies greater than 5%, with CYP2D6*1/*36 + *10 being the most common (11.46%). Tested variants, their positions and the genotype call for each of the samples are presented in Supplementary Table 1.

### *CYP2D6* next-generation sequencing

Sequencing reads were aligned to NG_008376.4. Of the 96 sequenced samples, one resulted in low coverage across the amplicon and was therefore removed from further analysis. The remaining 95 samples had an average read coverage of 851X across the region of interest. Average coverage per sample is provided in Supplementary Table 1. The TaqMan-based genotype calls presented in this table correspond to those published previously [29] and were confirmed with the sequencing data.

Overall, TaqMan-based genotype calls matched well with the NGS base calls with a few exceptions (Supplementary Table 1). Exceptions also included genotypes with a gene duplication or a *CYP2D7*–*2D6* hybrid, scenarios in which amplicon sequencing alone was not able to resolve or refute. All TaqMan-based genotype calls were confirmed, except for three samples: sample 1134146 was initially called CYP2D6*1/*10; sequencing, however, revealed that the CYP2D6*10-defining variants 100C >T and 4181G >C were homozygous for the variant allele (reference reads 0.64% and 0.00%, respectively), suggesting that the sample has a CYP2D6*10/*10 genotype. Sample 1142002 was originally called CYP2D6*10/*40 and was confirmed as harboring the defining variants for CYP2D6*10 (100C >T and 4181G >C), as well as having three of the four CYP2D6*40-defining variants (1022C >T, 2851C >T and 4181G >C); a manual check found no evidence for 1864-ins(TTTCGCCCC)2 in any of the samples but revealed the presence of 3202C >T, a unique SNP defining the CYP2D6*56 allele (in combination with 4181G >C). This SNP (3202C >T) was not identified by genotyping in any of the 211 samples, and except for 1142002 was not found in any other of the sequenced 95 samples. Last, sequencing was consistent with the presence of a CYP2D6*36 + *10 allele in sample 1111173, but the CYP2D6*100-defining SNP (2829delC) was not found, whereas 100C >T and 4181G >C, the other two SNPs of that haplotype, were confirmed. Review of the genotyping data revealed the presence of background amplification that was falsely interpreted as 2829delC being heterozygous, triggering the CYP2D6*100 call.

**Figure 1.**
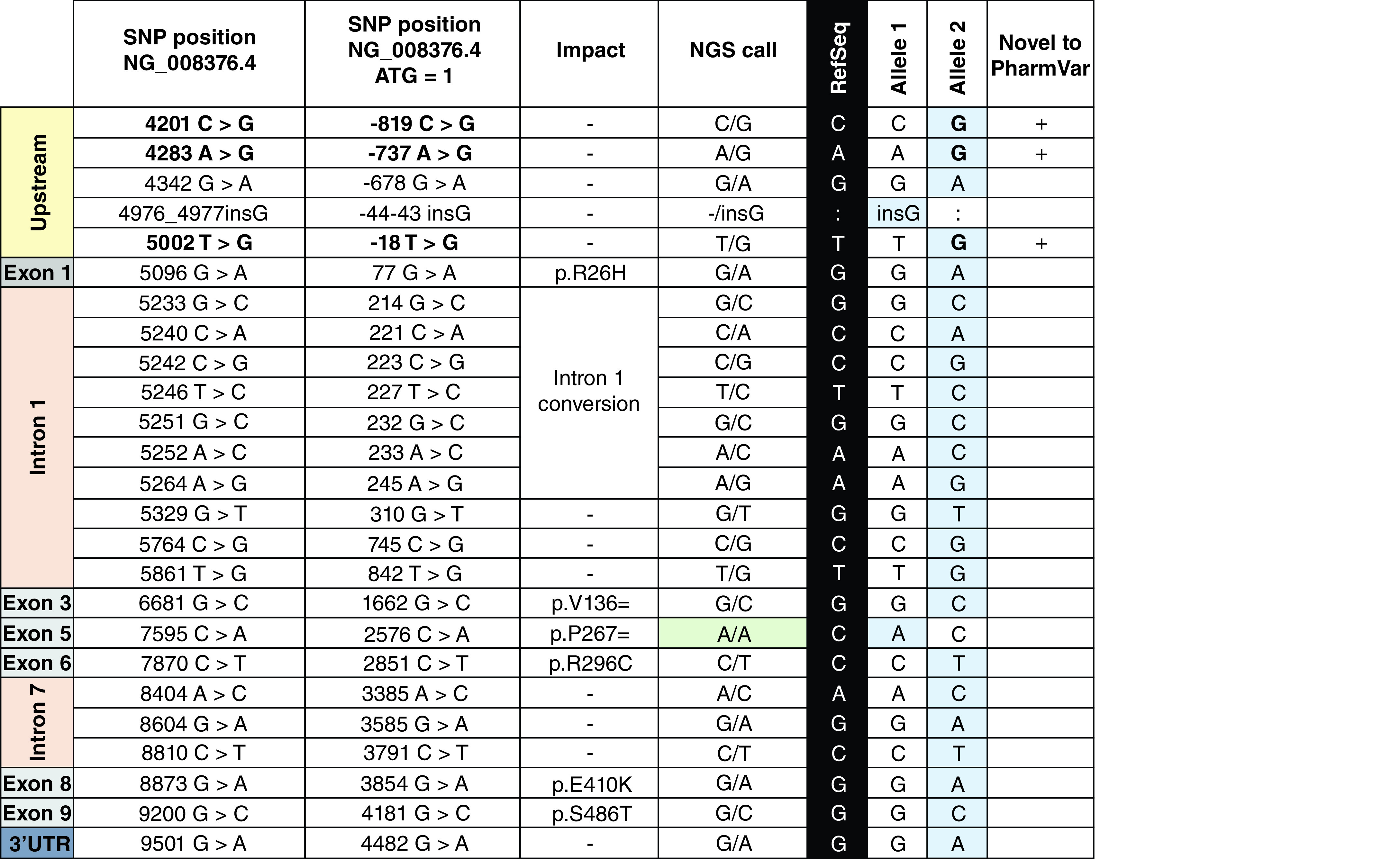
Summary of single-nucleotide polymorphisms discovered in sample 1040105 by next-generation sequencing and Sanger sequencing. ‘SNP position NG_008376.4’ denotes variant positions and nucleotide changes using NG_008376.4 as reference sequence counting from the sequence start. ‘SNP Position NG_008376.4 ATG = 1’ provides respective coordinates counting from the translation start codon (ATG = +1). ‘Impact’ indicates amino acid changes and the group of SNPs in intron 1 that are also commonly referred to as the ‘intron 1’ conversion (derived from *CYP2D7*). ‘RefSeq’ represents the reference nucleotide at that position (corresponding to that found in the CYP2D6*1.001 allele). ‘Allele 1’ and ‘Allele 2’ list the SNPs present on each of the two variant alleles as determined by Sanger sequencing of allele-specific amplicons; variant positions are highlighted by a blue background. SNPs in bold denote those new to PharmVar (i.e., have not been present in any other star alleles defined by PharmVar to-date). Alleles 1 and 2 were designated as CYP2D6*1.047 and *148.001. SNP: Single-nucleotide polymorphism.

In addition to assessing concordance between sequence and TaqMan-based genotype calls, the authors also determined whether any samples had alleles that eluded detection by TaqMan-based testing. Sample 1040105, originally genotyped as CYP2D6*1/*2, was confirmed harboring the defining variants for CYP2D6*2 (2851C >T and 4181G >C) but additionally revealed heterozygosity for 3854G >A, as well as several other SNPs, which could not be reconciled with haplotypes known to have this SNP (i.e., CYP2D6*27, *32 and *141). Allele-specific PCR and Sanger sequencing were performed to characterize each allele. Both alleles were found to harbor novel haplotypes and were submitted to PharmVar for allele designation. As shown in Figure 1, the novel CYP2D6*148 allele harbors 3854G >A in addition to several other variants; the second allele was designated as a novel suballele, CYP2D6*1.047.

In sample 1040105, 2576C >A was found to be homozygous for the variant allele by NGS (reference read 18.90%), whereas it was heterozygous by Sanger sequencing and found to be on the CYP2D6*1.047 suballele. In addition to this sample, the CYP2D6*1.047 suballele was inferred to be present in five other samples based on the NGS data. Owing to the lack of DNA for further analysis, the authors were unable to confirm the presence of the CYP2D6*1.047 suballele in any of the other samples by Sanger sequencing.

At the time of the analysis, a total of 319 variant positions between -1100 and 4482 were associated with annotated *CYP2D6* alleles in PharmVar. Across this region, 76 variant positions were identified across the Malagasy samples, of which 60 are annotated in PharmVar. The authors were interested in characterizing these 16 novel variant positions. Overall, these 16 variants were identified in a total of 23 samples. Of these variants, three were upstream of the coding start site, 10 were intronic, and three were exonic (Supplementary Table 2). Each of the 16 variants is reported to have an allele frequency of less than 0.25% in the Genome Aggregation Database (gnomAD, https://gnomad.broadinstitute.org/) (Supplementary Table 3). In the authors’ cohort, the frequencies ranged from 0.53% to 3.68% (Supplementary Table 2).

The upstream region, specifically a 160–170 bp stretch of nucleotides, is known to be difficult to resolve by Sanger sequencing due to the presence of A-tracks (see details in the Limitations & technical challenges section). Although these A-tracks should not cause misalignments in the case of NGS short reads, an unusually high number of SNPs were noticed in this and a further upstream region (n = 51). The authors believe these to be false-positive calls due to the high frequency in the samples (ranging from 0.53% to 62.11%) and historical precedence of misalignments in this genomic region. Therefore, the authors did not call variants within the -1600 and -1100 regions. Regarding the 10 intronic SNPs, 9 of these SNPs are well within the introns, whereas 1 (ATG start site 4034C >T) is within the boundary of intron 8 (ATG start site 4039) and exon 9 (ATG start site 4040). The three exonic variants were located at position 1607G >T (rs76060075) in exon 3, 2468G >A (rs867154253) in exon 5 and 3187A >C (rs141824015) in exon 7. The variants in exons 3 (sample 2090703) and 7 (sample 2070705) cause Gly118Val and Ile339Leu amino acid changes, respectively, whereas the variant in exon 5 (sample 1040709) is synonymous (Leu231=). The complete SNP profiles of these three samples are presented in Supplementary Table 4. Unfortunately, the presence of these exonic SNPs could not be confirmed by Sanger sequencing due to the lack of DNA.

## Discussion

Since the discovery of the polymorphic *CYP2D6* gene, it has been one of the most widely studied genes in the field of pharmacogenetics due to its direct role in the metabolism of many commonly prescribed medications [1]. Despite an extensive body of research over the past 30 years, interrogating *CYP2D6* variation has been proven to be challenging: *CYP2D6* genotyping assays are difficult to design and validate analytically because of the high number of known allelic variations and the presence of CNV and structural rearrangements between the *CYP2D6* and *CYP2D7* genes [3]. Furthermore, the nature and degree of *CYP2D6* variation in the population of interest may pose an issue; there may be no or only limited information available. Since commercially available platforms vary considerably in regard to sequence variations (or star alleles) and the extent of CNVs tested, unresolved discordances among genotyping platforms are a concern, especially when used clinically in populations with limited allele frequency data. Most of the commercially available platforms only interrogate the most commonly observed variants across populations. This, however, can be problematic, as rare population-specific variants may not be adequately identified. As a result, a patient’s predicted CYP2D6 metabolizer status, which is typically used for drug and/or dosing recommendations, may not be accurate [41,47].

Resequencing is the method of choice for validating SNP-based genotype results or for investigating and characterizing novel allelic variants, particularly in a new population. The *CYP2D6* gene is relatively easily resequenced by generating an XL-PCR product that encompasses the entire gene and subsequent sequence analysis. However, this approach reveals only sequence variations and does not inform phasing and haplotype structure, except in situations in which all SNPs are homozygous or only one SNP is heterozygous, or the allele of interest is paired with the CYP2D6*5 deletion allele or CYP2D6*13 (*CYP2D7*-*2D6*) hybrid structures, in which cases the desired allele is the only one amplified [46]. These limitations could potentially be resolved using third-generation sequencing technologies with longer read lengths and computational phasing; such approaches have been shown to inform phasing of SNPs over long distances and thus allow to more accurately determine a sample’s diplotype.

With a previous investigation of *CYP2D6* allelic variation in Madagascar by using commercially available TaqMan genotyping assays [29] supplemented with quantitative and qualitative CNV analyses, the authors sought in this study to validate those results and characterize rare or novel allelic variants in this unique admixed population by gene resequencing. The high coverage across the PharmVar variant positions provided confidence in the NGS-based genotyping calls. In 92 of 95 (96.8%) samples, TaqMan-based genotype calls matched the base calls from the sequencing. There were three exceptions: a sample that was genotyped as CYP2D6*1/*10 was found to be homozygous *10/*10 by sequencing. A sample genotyped as CYP2D6*10/*40 was found to carry 3202C >T, which is present in CYP2D6*56, whereas the 18-bp insertion of *40 was not detected by sequencing. Last, the presence of the CYP2D6*100 allele was not confirmed in a sample genotyped as CYP2D6*36 + *10/*100. Revisiting the original genotyping raw data led to the revision of the CYP2D*100 call. In this case, the false-positive call was triggered by an unusually high, likely nonspecific amplification; in contrast, the TaqMan assay results for the other two samples did not reveal any ambiguity. Due to the lack of DNA materials, the authors were not able to resolve these discordances. Since samples were recoded for genotyping, and genotyping and sequencing were not performed at the same time, it is possible that these samples were mislabeled. Resolving this issue in the authors’ future studies (see the following) is particularly important regarding the presence of the relatively rare nonfunctional CYP2D6*40 allele, which has been found in people of African ancestry, including African–Americans [52]. Although the CYP2D6*100 allele, which was originally discovered in Trinidadians of Indian ancestry [53], was not confirmed, it is not inconceivable that it occurs in the Malagasy considering the Malagasy genomic landscape: along with African and Austronesian connections, contributions from Arabic, Indian, Papuan and/or Jewish populations have been suggested [54].

Sequencing data also revealed that some SNPs can be present on allelic backgrounds other than those currently defined in the PharmVar database. SNP 3854G >A is part of the CYP2D6*27, *32 and *141 haplotype definitions but was not tested by genotyping, as no predesigned TaqMan assay is available. For sample 1040105, the authors were able to not only confirm the presence of the SNP by Sanger sequencing but also resolve the novel haplotype (Figure 1). Notably, the haplotype, designated CYP2D6*148 by PharmVar, comprises a novel combination of SNPs, all of which have been observed in other alleles. It remains uncertain though whether the amino acid changes present in this haplotype alter function and, if so, to what degree.

Finally, sequencing showed a total of 16 variant positions in the authors’ cohort that are not annotated in PharmVar. Among these were three exonic variants (Gly118Val in exon 3, Leu231= in exon 5 and Ile339Leu in exon 7) observed in one sample each. These exonic variants may be rare: Gly118Val was not found in any gnomAD population, whereas Leu231= and Ile339Leu were found only in African/African–American population at very low frequencies (Supplementary Table 3). Both samples with Gly118Val and Ile339Leu were genotyped as CYP2D6*36 + *10/*41; it remains unknown, however, whether these amino acid changes are located on the CYP2D6*10 or *41 allele (since the XL-PCR used for NGS does not amplify the *36 in the tandem arrangement, the authors tentatively exclude this allele having the novel variants). The synonymous SNP (Leu231=) was found in a sample genotyped as CYP2D6*1/*10, suggesting the presence of a novel CYP2D6*1 or *10 suballele. Interestingly, in addition to IM and PM phenotypes, some studies have shown that vivax relapses occurred in patients with CYP2D6*1/*10 and *2/*10 genotypes [55], which predict NM status. It is therefore tempting to speculate that Gly118Val and Ile339Leu, regardless of on which star allele they are located, may cause total loss of activity. As a result, these patients may bioactivate PQ much less effectively and consequently have a higher risk of relapsing. To resolve the haplotypes and substantiate the significance of these exonic variants, subjects in the authors’ ongoing investigation will be tested for these variants.

Regarding the 10 intronic SNPs, 9 are well within the introns, whereas 1 (4034C >T) is within the boundary of the intron 8/exon 9 splice junction. NetGene2-2.42, a server to predict intron splice sites in human [56,57], detected this splice site with a confidence score of 1.00. This result is in agreement with the previous finding of Rogan *et al.* [58] that there is a strong acceptor splice site at this location. Further analysis showed that 4034C >T does not reduce the level of confidence for the site. For validity purposes, the authors of this work tested NetGene2-2.42 for other known splice variants including those found in CYP2D6*4 (1847G >A) [58] and CYP2D6*41 (2989G >A) [59,60]. For CYP2D6*4, NetGene2-2.42 analysis indicated that 1847G >A inactivates splicing at the acceptor site and activates a new cryptic site one nucleotide downstream of the natural acceptor, with the confidence score reduced from 0.95 to 0.31. For CYP2D6*41, NetGene2-2.42 analysis predicted with borderline confidence (confidence score: 0.55) that there is a donor site, and 2989G >A increases the level of confidence for the site to 0.71. Given that these predictions are in line with what has been experimentally validated, 4034C >T is unlikely to impact splicing.

## Limitations & technical challenges

The authors acknowledge that this study has some limitations, and the results need to be validated in a larger population sample. *CYP2D6* interrogation and allele discovery has been performed using a variety of methodologies – targeted genotyping, allele-specific interrogation, Sanger sequencing, second-generation short-read sequencing and the recently developed third-generation long-read sequencing. Targeted genotyping platforms are widely used for research as well as in the clinical setting, which is driven by ease of use, low cost, accommodating low-to-medium sample throughput and only testing variants of known functional consequence. However, the limitations include potentially missing clinically relevant variants that were not genotyped, as well as out-of-date algorithms for genotype calling. Long-range PCR has facilitated more accurate assessment of the *CYP2D6* gene sequence; however, it has become clear that parallel copy number and sequence interrogation may be necessary to accurately call a patient’s *CYP2D6* diplotype and predict the metabolizer phenotype. In the present study, the presence of novel alleles and suballeles, the former attributed to 3854G >A being present on other allelic backgrounds than those currently defined in the PharmVar database, and novel exonic variants were discovered by gene resequencing. However, the NGS methodology, combined with manual checking of the sequence, did not find evidence for 1864-ins(TTTCGCCCC)2 in sample 1142002 (CYP2D6*10/*40). Identifying indels from NGS is known to be quite challenging and may be missed by second-generation short-read sequencing [61,62]. This issue and interference with the *CYP2D7* pseudogene, presence of CNVs and often complex structural rearrangements that are inherent to short-read *CYP2D6* sequencing have prompted the implementation of third-generation long-read sequencing of *CYP2D6* [63].

NGS revealed that in sample 1040105, 2576C >A was homozygous for the variant allele, whereas Sanger sequencing revealed that the SNP was heterozygous. There might be an explanation for this discrepancy, based on DNA secondary structures called 'G-quadruplexes' or 'G4s' [64]. G4s form within ssDNA, given the appropriate distribution of GC-rich motifs, and can physically block polymerase movement along DNA (especially when methylated), interfering with PCRs and other processes [65]. It has been noted that G4s can potentially impact *CYP2D6*, and 2576C >A lies within a predicted G4 sequence (Figure 1) [64]. That the 2576C >A genotype status may be affected by the presence of a predicted G4 sequence, and methylated cytosines, could help explain why this SNP appeared homozygous in NGS. To gain further insight regarding 2576C >A allelic dropout/imbalance, the authors plan to perform both NGS and Sanger sequencing on a larger number of samples in future investigations (see the following).

Another challenge that is faced when *CYP2D6* is sequenced are two A-tracks in the upstream region (-1258 to -1237 and -1103 to -1094), which may interfere with accurate PCR amplification. Essentially, the Taq polymerase may not accurately read through these A-tracks and add/delete A’s. In addition, the longer A-track is of variable length. Thus, the sequence in this region is often offset and at least with Sanger sequencing is difficult/impossible to read. Therefore, this region is consistently excluded from allele definitions by PharmVar. The authors were also unable to obtain unambiguous results for approximately 500 bp of the upstream region. Resolution of this region would require further investigation, including NGS of samples outside of Madagascar, to determine whether what was observed in this cohort is a true genomic region characteristic. Last, the sequencing data generated for this study cannot differentiate CYP2D6*36 + *10 from CYP2D6*10x2 alleles, although read ratios may be suggestive of the presence of a *10x2 gene duplication allele, as only the *10 gene copy of the *36 + *10 tandem is amplified. The reverse primer used to generate the XL-PCR template for sequencing does not bind to the *36 portion of a tandem due to the presence of *CYP2D7*-derived downstream sequences, and therefore no amplicon is produced. Of note, a ‘single’ CYP2D6*36 typically has a *CYP2D6* downstream region and thus would be amplified, and subsequent sequencing would reveal a series of SNPs in exon 9 which are, due to their *CYP2D7* origin, known as exon 9 conversion. The CYP2D6*36 + *10 allele calls are based on the authors’ previous study that utilized quantitative and qualitative methods to determine CNV status.

## Future studies

The authors have recently launched a study where a much larger sample size will be analyzed for *CYP2D6* variation by utilizing a range of methods including TaqMan-based genotyping, NGS-based gene sequencing and quantitative CNV analysis, as well as bioinformatic tools (e.g., Aldy, Stargazer, StellarPGx and/or Astrolabe), to determine *CYP2D6* genotype. The purpose of this new investigation is to capture *CYP2D6* variation as completely as possible, then to perform genetic association analyses with PQ metabolism and efficacy against vivax malaria. The gene sequencing will be used to resolve any discordant genotype to phenotype findings. An emphasis would be on the new exonic variants, not only on their frequencies but also on their potential functional significance. Given that a substantial proportion of the Malagasy population has decreased CYP2D6 activity, and there is a unique genetic admixture of *CYP2D6* [29], examining the functional significance of any new exonic variant is imperative. The potential functional significance of the three new upstream SNPs would also be pursued in future studies.

## Conclusion

In the present study, using one of the NGS platforms, a high concordance between prior TaqMan-based *CYP2D6* genotype calls and the base calls from sequencing was observed. In addition, the study revealed that there are new variants and haplotypes present in the Malagasy. These findings strongly support the value of sequencing unique admixed populations to gain more detailed and accurate insights regarding pharmacogene variability. A more comprehensive understanding of *CYP2D6* variation will help us better understand and ultimately predict interindividual PQ response, especially in the Malagasy and other admixed populations, where vivax malaria is a complex clinical and public health problem.

**Summary points**

- Among the large number of drugs that undergo CYP2D6-mediated metabolism is an 8-aminoquinoline antimalarial primaquine (PQ), a drug of choice against latent malaria caused by *Plasmodium vivax*.
- Recent clinical and laboratory evidence suggest that the efficacy of PQ may depend on genetic variation in *CYP2D6* – PQ treatment failures have predominantly been observed in patients predicted to be intermediate and poor metabolizers.
- Recently, for the first time, the authors investigated *CYP2D6* genetic variation in Madagascar, where vivax malaria is endemic. In addition, the Malagasy populations offer a unique, rich admixture history between Southeast Asian and East African ancestral populations.
- In the present study, the authors applied a next-generation sequencing approach to further characterize the *CYP2D6* variation in Madagascar and compare it with the TaqMan™-based genotype data.
- The authors observed high concordance between TaqMan-based *CYP2D6* genotype calls and the base calls from sequencing. In addition, this study revealed that there are new variants and haplotypes present in the Malagasy.
- These findings strongly support the value of sequencing unique admixed populations to gain more detailed and accurate insights regarding pharmacogene variability.
- A more comprehensive understanding of *CYP2D6* variation will not only benefit successful *P. vivax* treatment, and eventual elimination, in Madagascar but all populations afflicted by this insidiously harmful infection.
